# Use of an electronic portfolio for longitudinal assessment of personal and professional development in undergraduate medical education

**DOI:** 10.3389/fmed.2024.1505378

**Published:** 2025-01-06

**Authors:** Michelle Schmude, Ian McCoog, Tanja Adonizio, Halle B. Ellison, Alison Howe, Amanda M. Caleb

**Affiliations:** ^1^Department of Medical Education, Geisinger College of Health Sciences, Scranton, PA, United States; ^2^Palliative Care, Geisinger Health, Danville, PA, United States

**Keywords:** professionalism, ePortfolio, medical education, assessment, personal development, curriculum, program outcome

## Introduction

### Assessing the professionalism competency in undergraduate medical education

Contemporary medical education is increasingly competency-based and requires a curricular framework to support individual students' professional identity formation concurrently with defined and benchmarked competencies (1, 2). The transition to a competency-based curriculum requires both longitudinal and integrated assessments (Schmude et al., unpublished),^1^ which are challenging to deliver on an individual level (2–4). Much has been written regarding the evaluation of professionalism, especially among medical students and residents (5). Expert consensus about professionalism assessment is that “Professionalism should be assessed longitudinally. It requires combinations of different approaches, assessing professionalism at individual, interpersonal, and societal/institutional levels” (6). The challenge to medical educators in the longitudinal assessment of professionalism involves integrating a large and dispersed amount of data into an organized and easily accessible format (7, 8, 29, 30). Our work focuses on promoting adult learners' professional identity formation through a longitudinal Personal and Professional Development curriculum in undergraduate medical education.

### ePortfolios in higher education and medical education

ePortfolios have been identified as a high-impact practice for education and are widely used by colleges and universities in the United States (9). Developing an ePortfolio allows adult learners to cultivate their skills as master learners and participate actively in their education, a key tenet of adult learning theory (11). The Association of American Colleges and Universities has worked to promote the value of portfolios in assessing student learning through their Valid Assessment of Learning in Undergraduate Education (VALUE) project (https://www.aacu.org/initiatives/value). Scholars who study student assessments have evaluated ePortfolios to promote and assess student learning (7, 9). These researchers similarly conclude that professionalism is not a solitary pursuit; student self-reflection and self-assessment without external guidance are suboptimal (12). Faculty increasingly realize the value of implementing ePortfolios for students to demonstrate competence, while students appreciate the value of reflecting on what they learn throughout their medical education (9). Researchers have reported that ePortfolios promote students' reflection and learning: one group reported that “ePortfolios are currently celebrated as a way to facilitate and document more authentic forms of assessment” (13, 14). Subsequent studies have reported positive student perceptions of using portfolios, particularly the utility of reflective learning (15–17).

Although ePortfolio use in medical education continues to grow, documented use within a medical school curriculum is still developing (10, 18). Early descriptions of ePortfolio use included how portfolios could be utilized in medical education (7, 8, 30), yet few medical schools have used ePortfolios to assess professionalism.

### Our goals for incorporating an ePortfolio

At Geisinger Commonwealth School of Medicine (GCSOM), we defined the ePortfolio as a student-assembled electronic portfolio that displays academic accomplishments, essays, videos, samples of work, and volunteer and extracurricular activities in required and optional, student-driven artifacts. This tool can monitor and promote each student's curricular progress through course assignments tied to these ePortfolio artifacts related to competency development (11, 19). Comprehensive ePortfolio assessment data for Doctor of Medicine (MD) program evaluation can highlight opportunities for continuous quality improvement. Therefore, developing and implementing an ePortfolio may be applied to any competency-based educational setting, such as health professions and medical education (16).

We felt that using an ePortfolio to assess professionalism could have several benefits, including (1) enabling multidimensional assessment by facilitating longitudinal integration of students' assignments, termed artifacts, (2) highlighting opportunities for continuous quality improvement within our curriculum and (3) serving as an example for educators using an ePortfolio in any competency-based educational setting. Therefore, we aim to share our experience with developing and implementing an ePortfolio for longitudinal assessment of professionalism within undergraduate medical education.

## Methods

### Development and implementation of the ePortfolio

The GCSOM Total Health Curriculum includes personal and professional development as one of the six programmatic objectives woven throughout the MD curriculum (Appendix A). The personal and professional development curriculum, one of six longitudinal curricular themes, is used to demonstrate achievement in meeting the learning objectives of the MD curriculum (see text footnote 1). The development and implementation of the ePortfolio assessment at GCSOM was based on Design Thinking, which incorporates organizational and systems theory with social psychology and practical research through inspiration, ideation, and implementation (20). By reflecting on their learning, students practiced collective generation of ideas, rapid prototyping, and continuous testing to align technologically based tools with users' needs (20). In this manner, we engaged student leaders and faculty champions to review the implementation and provide timely feedback for improving the ePortfolio (21). Partnering with a faculty member, students engaged in ePortfolio development while strengthening their professional identity formation (20, 22, 23).

To begin the implementation of the ePortfolio, we evaluated the curriculum to identify assessments that would be appropriate to use as evidence of individual student growth and achievement in the curriculum's personal and professional development programmatic learning objectives (10, 24). The step-by-step inventory process included:

1. Assemble faculty members responsible for assessment within the MD curriculum.
2. Create an inventory of MD curricular assessments by course and class session.
3. Identify gaps in MD curricular assessments related to the programmatic learning objectives that the ePortfolio could address.
4. Propose assessments to address the gaps to meet programmatic learning objectives.
5. Link and map identified assessments to the programmatic learning objectives.
6. Create labels for ePortfolio evidence to track students' growth in reflection and professionalism.

Three GCSOM medical students developed several ePortfolio prototypes. One was selected based on its ability to track students' progress toward achieving programmatic learning objectives (25).

### Current processes and continuous quality improvement for the ePortfolio

All first-year students attend a session that explains the programmatic requirements, introduces the personal and professional development curriculum, and provides an overview of the ePortfolio. Students are also given an ePortfolio manual so they have the necessary educational guidance to construct their ePortfolio correctly. In addition, an active learning workshop is conducted so students can build confidence in using the ePortfolio within the Portfolium platform and upload evidence in real time while receiving feedback from faculty members.

The longitudinal personal and professional development ePortfolio was created in Portfolium (https://portfolium.com) and embedded as a curricular requirement for all students. Students completed ePortfolio entries for corresponding assignments that reinforced what they learned in basic science and doctoring courses and themes related to professionalism, social justice, health equity, and community engagement (19, 25). Personal and Professional Development theme faculty members evaluated student submissions, termed evidence, within the ePortfolios and provided feedback regarding student growth in the personal and professional development curriculum. These faculty members also mentored students' reflective skills and construction of the ePortfolio. In addition, courses throughout the MD curriculum support the student's growth for reflective practice. Students submitted data were used to determine if curricular requirements were achieved.

Reflective writing and practices are embedded throughout the MD curriculum, providing formative feedback to students for future incorporation into their learning. Students were given a document that contained the specific ePortfolio evidence needed by class year, assignment name, name to be used in Portfolium for the type of evidence, and the specific labels to assign to the evidence. ePortfolio labels can be assigned at the individual student level (personal use), theme level (evidence for the personal and professional development theme), and programmatic level (evidence for programmatic objectives). Labels allowed for ePortfolio assessment data to be used by faculty in institutional research for MD program evaluation and to identify opportunities for continuous quality improvement. Students were periodically reminded via announcements in the Canvas Learning Management System (www.instructure.com/canvas) about ePortfolio deadlines and requirements. At the end of the academic year, students were required to upload their completed ePortfolio from Portfolium into a designated assignment in Canvas for individual student evaluation by faculty.

One example of an educational activity within the personal and professional development theme is viewing the Association of American Medical College virtual seminar Legacy of Medicine in the Holocaust. After watching the seminar, students must complete a reflective writing assignment, which they are required to submit as an ePortfolio assignment. The reflective writing submission links directly to MD program outcomes and is contained as evidence within the ePortfolio. The associated ePortfolio “skills” document the MD program outcomes directly related to the students' achieved competency (26).

## Results

Each required personal and professional development curriculum assignment for the ePortfolio has labels, known within Portfolium as *skills*, that are linked back to the MD programmatic objectives. Creating labels allows for the tracking of individual student growth and assessing the personal and professional development curriculum. These results can be tracked at the individual student level annually and longitudinally. Annual curricular review allows faculty and students to evaluate the breadth of program objectives covered in that year's assignments. The data contained within the ePortfolio can be used to generate theme content maps relevant to each phase of the curriculum to address particular educational program objectives and provide precision education to enhance student learning and outcomes.

Faculty are trained to use the REFLECT rubric and Began Model (27, 28) to assess assigned reflections within the ePortfolio. These assessment tools (27, 28) provide a standardized process for evaluating students' reflections and providing formative feedback regarding their achievement of learning outcomes (see text footnote 1). Based on the individual assessment of student learning throughout the MD program, artifacts contained within the ePortfolio linked to longitudinal student learning, and the skills associated with achieving MD programmatic outcomes, we believe the ePortfolio achieved the goals.

## Discussion

In 2021, GCSOM expanded its use of the data warehousing and visualization software One45 Analytics. The Analytics modules included data related to prematriculation, student performance on local assessments, and nationally normed gateway exams. One45 Analytics software modules allowed GCSOM staff to access and label curricular data points for more detailed tracking and visualization on data dashboards. MD program objectives and personal and professional development curricula can be labeled in Canvas, and data that flow from Canvas assignments into Analytics can thus be used for data-driven decision-making. Assignments completed in fulfillment of the personal and professional development curriculum, including success in the ePortfolio, can be identified so that a student's academic advisor can intervene if there are concerns about the student's progress in this area of the curriculum.

In addition, ePortfolio entries created as Canvas assignments allow students to label their posts with the appropriate program objective and personal and professional development curriculum assignment. In this manner, ePortfolios create a collection of academic artifacts for students and concurrently display a diversified array of skills that can be valuable during residency interviews. Standardized labels can streamline communication between students and faculty; however, students can create their own labeling schema for the personal and professional development curriculum use. This allows students to showcase special skills or experiences that they feel can contribute significantly to the residency programs they apply to.

The next steps for our ePortfolio initiative include optimizing Analytics reporting capabilities and further staff development. For example, GCSOM hopes to give course directors access to course-specific data and faculty advisors access to their advisees' data. Currently, all data are requested by faculty and then collected, synthesized, and disseminated by the Office of Institutional Research, facilitated by the ability to create standard reports within Analytics. Granting course directors and faculty advisors this level of access requires refined data tables to ensure that faculty can view only assessment data related to a student's performance in the faculty member's purview and not the student's overall record of achievement, thus avoiding a potential source of bias in assessment.

As access evolves during the maturation of ePortfolio use, the need for faculty development will increase. Concerning One45 Analytics, we will implement a *train-the-trainer* model starting with those who work closely with the curriculum, academic progress, and assessment data, progressing to course leadership, and then to other faculty and staff as necessary. Faculty and staff who regularly use Portfolium will share their successes and promote using the ePortfolio product in tracking MD programmatic requirements while providing precision education to learners. The ePortfolio has become an essential part of the longitudinal assessment of the personal and professional development curriculum and students' performance throughout the curriculum as a whole. By integrating numerous systems and assessment types, GCSOM can create a more holistic picture of student performance that benefits not only the institution's ability to report accurate data but, more importantly, helps students reflect upon their experiences in a way that guides them in taking the next step in their careers.

## Figures and Tables

**Figure A1:**
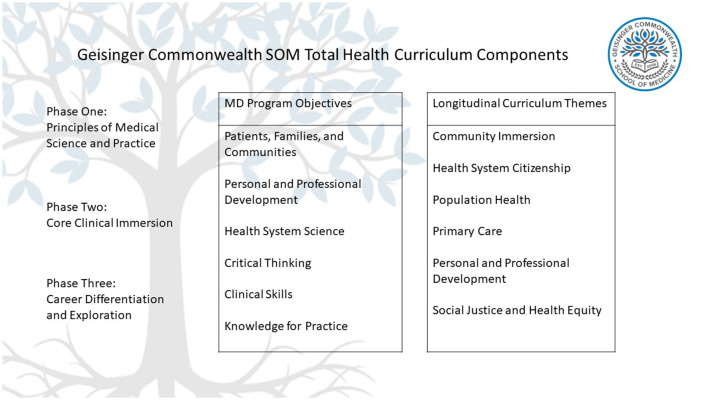
Geisinger commonwealth SOM total health curriculum components.

## Footnotes

^1^Schmude, M., Adonizio, T., Ellison, H. B., Weber, R., Arnott, J., and Koestler, J. (unpublished). “Teaching Professionalism,” in *Medical Education and Fostering Professionals in Medicine*, eds. A. Fornari and N. Deming (New York, NY: Springer).
